# Impact of intracellular toxic advanced glycation end-products (TAGE) on murine myoblast cell death

**DOI:** 10.1186/s13098-020-00561-z

**Published:** 2020-06-29

**Authors:** Takanobu Takata, Akiko Sakasai-Sakai, Masayoshi Takeuchi

**Affiliations:** Department of Advanced Medicine, Medical Research Institute, Kanazawa Medical University, Uchinada-machi, Ishikawa, 920-0293 Japan

**Keywords:** Sarcopenia, Lifestyle-related diseases, Myoblasts, Advanced glycation end-products, Glyceraldehyde, Toxic advanced glycation end-products, C2C12 cells

## Abstract

**Background:**

Sarcopenia is a progressive condition that is characterized by decreases in skeletal muscle mass and function. Although sarcopenia is associated with lifestyle-related diseases (LSRD), the mechanisms underlying cell death in myoblasts, which differentiate to myotubes, remain unclear. We previously designated glyceraldehyde (an intermediate of glucose/fructose metabolism)-derived advanced glycation end-products (AGEs) as toxic AGEs (TAGE) because of their cytotoxicity and involvement in LSRD, and hypothesized that TAGE contribute to cell death in myoblasts.

**Methods:**

C2C12 cells, which are murine myoblasts, were treated with 0, 0.5, 1, 1.5, and 2 mM glyceraldehyde for 24 h. Cell viability and intracellular TAGE were then assessed using 5-[2,4,-bis(sodioxysulfonyl)phenyl]-3-(2-methoxy-4-nitrophenyl)-2-(4-nitrophenyl)-2*H*-tetrazole-3-ium (WST-8) and slot blot assays. Cells were pretreated with 8 mM aminoguanidine, an inhibitor of AGE production, for 2 h, followed by 0, 1.5, and 2 mM glyceraldehyde for 24 h. Cell viability and intracellular TAGE levels were then assessed. Serum TAGE levels in STAM mice, in which there were four stages (no steatosis, simple steatosis, steatohepatitis, and fibrosis), were measured using a competitive enzyme-linked immunosorbent assay. Results were expressed as TAGE units (U) per milliliter of serum, with 1 U corresponding to 1.0 μg of glyceraldehyde-derived AGE-bovine serum albumin (BSA) (TAGE-BSA). The viability of cells treated with 20, 50, and 100 μg/mL non-glycated BSA and TAGE-BSA for 24 h was assessed using the WST-8 assay.

**Results:**

In C2C12 cells treated with 1.5 and 2 mM glyceraldehyde, cell viability decreased to 47.7% (*p *= 0.0021) and 5.0% (*p *= 0.0001) and intracellular TAGE levels increased to 6.0 and 15.9 μg/mg protein, respectively. Changes in cell viability and TAGE production were completely inhibited by 8 mM aminoguanidine. Serum TAGE levels at the steatohepatitis and fibrosis stages were 10.51 ± 1.16 and 10.44 ± 0.95 U/mL, respectively, and were higher than those at the no steatosis stage (7.27 ± 0.18 U/mL). Cell death was not induced by 20 or 50 μg/mL TAGE-BSA. The viabilities of C2C12 cells treated with 100 μg/mL non-glycated BSA and TAGE-BSA were 105.0% (*p *= 0.2890) and 85.3% (*p *= 0.0217), respectively.

**Conclusion:**

Intracellular TAGE strongly induced cell death in C2C12 cells and may also induce myoblast cell death in LSRD model mice.

**Electronic supplementary material:**

The online version of this article (doi:10.1186/s13098-020-00561-z) contains supplementary material, which is available to authorized users.

## Background

Skeletal muscle requires exercise, the synthesis of glycogen, and interactions with other organs, such as the liver and adipose tissue [1]. Sarcopenia is a progressive condition that is characterized by decreases in skeletal muscle mass and function, resulting in the deterioration of activities of daily living and quality of life as well as increases in the risk of falls and mortality [2]. Skeletal muscle consists of myotubes that differentiate from myoblasts; therefore, one of the mechanisms contributing to the loss of skeletal muscle is the death of or dysfunctions in myoblasts [2–6]. Accumulating evidence has shown that lifestyle-related diseases (LSRD) such as type 2 diabetes mellitus (T2DM) and non-alcoholic steatohepatitis (NASH) has an increased risk of sarcopenia [2, 7, 8]. However, the mechanisms underlying cell death in myoblasts in LSRD model animals and patients with LSRD have not yet been elucidated. The relationships between cell death in myoblasts in vitro and risk factors for LSRD currently remain unclear. Mastrocola et al. previously reported that the levels of *N*^ε^-carboxymethyllysine (CML) and *N*^ε^-carboxyethyllysine (CEL)-modified proteins, which are advanced glycation end-products (AGEs), were elevated in the skeletal muscle of C57Bl/6j mice and ob/ob mice, which is an obese model mouse, and identified abnormalities in skeletal muscle (including the loss of skeletal muscle mass, myosteatosis, and oxidative stress) [9, 10]. CML and CEL-modified proteins have been suggested to play a role in sarcopenia. Although the relationships between these AGEs and cell death in myoblasts remain unclear, based on the findings reported by Mastrocola et al. from skeletal muscle tissue, AGEs may be generated in myoblasts.

In the present study, we investigated whether glyceraldehyde (an intermediate of glucose/fructose metabolism)-derived AGEs were generated in C2C12 cells, which are murine myoblasts. We focused on glyceraldehyde because we previously designated glyceraldehyde-derived AGEs as toxic AGEs (TAGE) based on their cytotoxicity and involvement in LSRD, such as T2DM, NASH, cardiovascular diseases (CVD), and cancer [11–15]. TAGE appear to be generated in myoblasts and induce cytotoxicity based on previous findings showing their production in neuroblastoma cells, hepatic cells, pancreatic cells, and cardiac cells as well as their induction of cell death and dysfunction [16–22]. TAGE in the blood which are one of extracellular TAGE increase in patients with LSRD and induce cytotoxicity in cells via receptor for AGEs (RAGE) [11–13, 15, 23]. Extracellular TAGE which are generated in other cells and secreted or released into the blood may induce cytotoxicity in myoblasts because they express RAGE [2]. Therefore, we investigated the cytotoxicity of glyceraldehyde-derived AGE-bovine serum albumin (BSA) (TAGE-BSA), a model of extracellular TAGE, in C2C12 cells.

## Methods

### Reagents, cell lines, and serum of STAM mice

Dulbecco’s modified Eagle’s medium (DMEM) and penicillin–streptomycin solution were obtained from Sigma-Aldrich (MO, USA). Fetal bovine serum (FBS) was purchased from Bovogen-Biologicals (VIC, Australia). Glyceraldehyde was purchased from Nacalai Tesque Inc. (Kyoto, Japan). The 5-[2,4,-Bis(sodioxysulfonyl)phenyl]-3-(2-methoxy-4-nitrophenyl)-2-(4-nitrophenyl)-2*H*-tetrazole-3-ium (WST-8) assay kit and 3-[(3-cholamido-propyl)-dimethyl-ammonio]-1-propane sulfonate) (CHAPS) were obtained from Dojindo Laboratories (Kumamoto, Japan). The ethylene diamine-N,N,N’,N’-tetraacetic acid (EDTA)-free protease inhibitor cocktail was obtained from Roche Applied Science (Penzberg, Germany). C2C12 cells were obtained from KAC Co., Ltd. (Kyoto, Japan). The serum of non-fasted STAM mice was purchased from SMC Laboratories, Inc. (Tokyo, Japan). The protein assay kit for the Bradford method was obtained from Takara Bio, Inc. (Otsu, Japan). A horseradish peroxidase (HRP)-linked molecular marker was obtained from Bionexus (CA, USA). A HRP-linked goat anti-rabbit IgG antibody was purchased from DAKO (Glostrup, Denmark). All other reagents and kits not indicated were purchased from Fujifilm Wako Pure Chemical Co. (Osaka, Japan). TAGE-BSA, non-glycated BSA, and an anti-TAGE antibody were prepared as described previously [24].

### Cell culture and cell seeds

C2C12 cells were incubated in DMEM supplemented with 10% FBS, 100 U/mL penicillin, and 100 mg/mL streptomycin under standard cell culture conditions (humidified atmosphere, 5% CO_2_, 37 °C). Cells were seeded (1.9 × 10^4^ cells/cm^2^) on 96-well microplates and culture dishes (Becton–Dickinson, NJ, USA).

### Glyceraldehyde and aminoguanidine treatments of C2C12 cells

Glyceraldehyde was dissolved in phosphate-buffered saline (PBS) without Ca^++^ and Mg^++^ ((PBS)(−)), and then filtered before being added to C2C12 cells. The volume of PBS (−) (including glyceraldehyde) was 2.0 μL/100 μL of the total medium volume. All experiments were performed 24 h after treatments with 0, 0.5, 1, 1.5, and 2 mM glyceraldehyde. The cell culture method before the treatment with aminoguanidine (the volume of PBS (−) (including aminoguanidine) was 2.0 μL/100 μL of the total medium volume), an inhibitor of AGE production, was the same as that described above. Cells were pretreated with 0 or 8 mM aminoguanidine for 2 h followed by 0, 1.5, and 2 mM glyceraldehyde for 24 h (the volume of PBS(−) (including glyceraldehyde) was 2.0 μL/102 μL of the total medium volume).

### Cell viability of C2C12 cells treated with glyceraldehyde and aminoguanidine

Cell viability was assessed using the WST-8 assay. Medium containing glyceraldehyde/aminoguanidine was removed and cells were washed with PBS (−). Ten microliters of WST-8 reagent was added to 96-well microplates in which C2C12 cells were cultured in medium (100 μL), and this was followed by an incubation at 37 °C for 2 h in a CO_2_ incubator. Absorbance was measured at 450 and 655 nm using a microplate reader (Bio-Rad, CA, USA). Medium in the wells without cells was treated with glyceraldehyde/aminoguanidine, a medium change, and WST-8 reagent to measure background absorbance. Background absorbance was subtracted from experimental values.

### Assessment of intracellular TAGE in C2C12 cells treated with glyceraldehyde and aminoguanidine using a slot blot analysis

This analysis was performed as described previously with some modifications [20–22]. Cells were washed with (PBS)(−) and then lysed in buffer [a solution of 2 M thiourea, 7 M urea, 4% CHAPS, and 30 mM Tris, and a solution of EDTA-free protease inhibitor cocktail (9:1)]. Cell extracts were then incubated on ice for 20 min, centrifuged at 10,000×*g* at 4 °C for 15 min, and the supernatant was collected as the cell extract. Protein concentrations were measured using the protein assay kit for the Bradford method with BSA as a standard. Regarding the detection of TAGE, equal amounts of cell extracts, the HRP-linked molecular marker, and TAGE-BSA were loaded onto polyvinylidene difluoride (PVDF) membranes (0.45 μm; Millipore, MA, USA) fixed in the slot blot apparatus (Bio-Rad). PVDF membranes were cut to prepare two membranes and then blocked at room temperature (r.t.) for 1 h using 5% skimmed milk in PBS(−) containing 0.05% Tween 20 (skimmed milk-PBS-T). After this step, we used 0.5% of skimmed milk-PBS-T for washing or as the solvent of antibodies. After washing twice, membranes were incubated with (1) the anti-TAGE antibody (1:1000) or (2) neutralized anti-TAGE antibody (a mixture of the anti-TAGE antibody (1:1000) and 250 µg/mL of TAGE-BSA) at 4 °C overnight. Membranes were then washed four times. Proteins on the membrane were incubated with the HRP-linked goat anti-rabbit IgG antibody (1:2000) at r.t. for 1 h. After washing three times with PBS-T, membranes were moved into PBS(−). Immunoreactive proteins were detected with the ImmunoStar LD kit and band densities on the membranes were measured using the Fusion FX fluorescence imager (M&S Instruments Inc., Osaka, Japan). The densities of HRP-linked molecular marker bands were used to correct for differences in densities between membranes. The amount of TAGE in cell extracts was calculated based on a calibration curve for TAGE-BSA.

### Analysis of serum TAGE levels in STAM mice

TAGE levels in the serum of STAM mice were measured using a competitive enzyme-linked immunosorbent assay. The serum of mice, in which stages were no steatosis, simple steatosis, steatohepatitis, and fibrosis (four mice in each group) was analyzed. Briefly, each well of the 96-well microplate was coated with 1.0 μg/mL TAGE-BSA and incubated overnight in a cold room. Wells were washed three times with 0.3 mL of PBS containing 0.05% Tween 20 (PBS-T). Wells were then blocked by an incubation for 1 h with 0.2 mL of a solution of PBS containing 1% BSA. After washing with PBS-T, test samples (50 µL) were added to each well as a competitor for 50 µL of the anti-TAGE antibody (1:1000), followed by an incubation at r.t. for 2 h with gentle shaking on a horizontal rotary shaker. Wells were then washed with PBS-T and developed with alkaline phosphatase-linked anti-rabbit IgG utilizing *p*-nitrophenyl phosphate as the colorimetric substrate. Results were expressed as TAGE units (U) per milliliter of serum, with 1 U corresponding to 1.0 μg of a TAGE-BSA standard as described previously [24]. Sensitivity and intra- and interassay coefficients of variation were 0.01 U/mL and 6.2 and 8.8%, respectively [25].

### Non-glycated BSA and TAGE-BSA treatment of C2C12 cells and assessment of cell viability

C2C12 cells were treated with 0, 20 50, and 100 μg/mL of non-glycated BSA and TAGE-BSA, and then incubated for 24 h. Cell viability was measured using the WST-8 assay. The ratio of cell viability was calculated based on the viability of cells treated with TAGE-BSA versus those treated with non-glycated BSA.

### Statistical analysis

Stat Flex (ver. 6) software (Artech Co., Ltd., Osaka, Japan) was used for statistical analyses. Data were expressed as mean ± standard deviation (S.D.). When statistical analyses were performed on data, significant differences in the means of each group were assessed by a one-way analysis of variance (ANOVA). We then used the Bonferroni or Tukey’s test for an analysis of variance. P-values < 0.05 were considered to be significant.

## Results

### Viability of C2C12 cells treated with glyceraldehyde

**Fig. 1:**
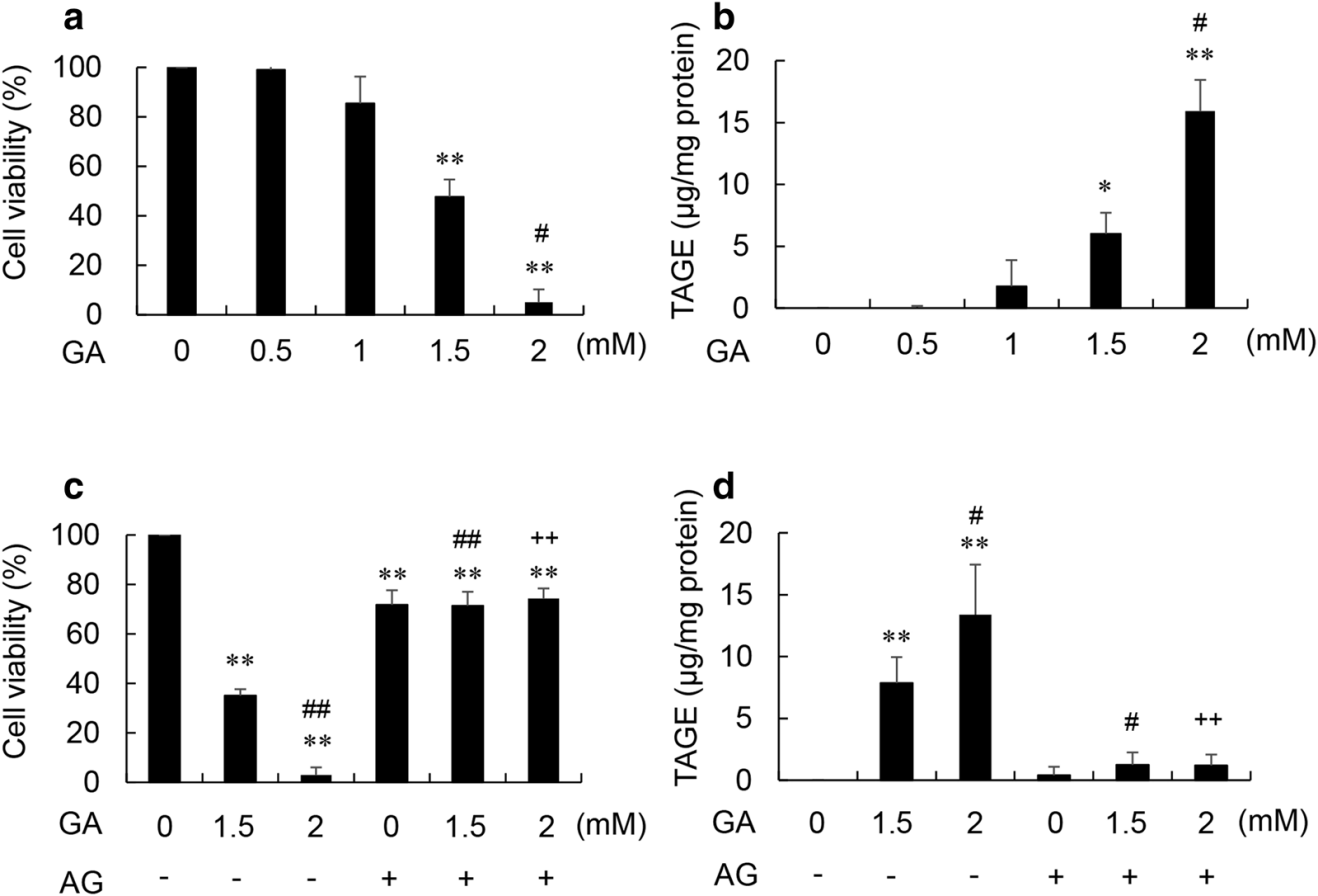
Cell viability and intracellular TAGE levels in C2C12 cells treated with glyceraldehyde and aminoguanidine. GA: glyceraldehyde. AG: aminoguanidine. **a**, **b** Cells were treated with 0, 0.5, 1, 1.5, and 2 mM GA for 24 h. **c, d** Cells were pretreated with 0 or 8 mM AG for 2 h, followed by 0, 1.5, and 2 mM GA for 24 h. **a, c** Cell viability was assessed by the WST-8 assay, which was performed in three independent experiments. One experiment was performed using 7 wells to calculate the average. Data are shown as mean ± S.D. (N = 3). **b**, **d** Intracellular TAGE were analyzed using a slot blot analysis. Cell lysates (2.0 μg of protein/lane) were blotted onto a polyvinylidene difluoride membrane. The densities of HRP-linked molecular marker bands were used to correct for differences in densities between membranes. The amount of TAGE was calculated based on a calibration curve for TAGE-BSA. A slot blot analysis was performed in three independent experiments. One experiment was performed using 2 lanes to calculate the average. Data are shown as mean ± S.D. (N = 3). **a**, **b** P-values were based on the Bonferroni test. ^*^p < 0.05 vs. 0 mM GA. ^**^p < 0.01 vs. 0 mM GA. ^#^*p *< 0.05 vs. 1.5 mM GA. **c**, **d** P-values were based on Tukey’s test. ^**^p < 0.01 vs. 0 mM GA without AG. ^#^*p *< 0.05 vs. 1.5 mM GA without AG. ^##^*p *< 0.01 vs. 1.5 mM GA without AG. ^++^*p *< 0.01 vs 2 mM GA without AG

The viability of C2C12 cells treated with 0.5 and 1 mM glyceraldehyde did not decrease, whereas dose-dependent decreases to 47.7 and 5.0% were observed in those treated with 1.5 and 2 mM glyceraldehyde, respectively (Fig. 1a).

### Quantity of intracellular TAGE in C2C12 cells treated with glyceraldehyde

Intracellular TAGE were not generated in C2C12 cells treated with 0, 0.5, and 1 mM glyceraldehyde (Fig. 1b and Additional file 1: Fig. S1a). Intracellular TAGE dose-dependently increased to 6.0 and 15.9 μg/mg protein in C2C12 cells treated with 1.5 and 2 mM glyceraldehyde, respectively (Fig. 1b and Additional file 1: Fig. S1a).

### Effects of the aminoguanidine pretreatment on the viability of C2C12 cells treated with glyceraldehyde

The viability of C2C12 cells treated with 1.5 and 2 mM glyceraldehyde without aminoguanidine dose-dependently decreased to 35.0 and 3.0%, respectively (Fig. 1c). In C2C12 cells pretreated with 8 mM aminoguanidine, cell viabilities were 71.7, 71.3, and 74.3% in those subsequently treated with 0, 1.5, and 2 mM glyceraldehyde, respectively. No significant differences were observed between each treatment (Fig. 1c). The aminoguanidine pretreatment completely inhibited decreases in the viability of C2C12 cells treated with 1.5 and 2 mM glyceraldehyde.

### Effects of the aminoguanidine pretreatment on the quantity of intracellular TAGE in C2C12 cells treated with glyceraldehyde

Glyceraldehyde concentrations of 1.5 and 2 mM without aminoguanidine dose-dependently increased intracellular TAGE to 7.9 and 13.4 μg/mg protein, respectively (Fig. 1d and Additional file 1: Fig. S1b). Intracellular TAGE levels in C2C12 cells pretreated with 0 mM aminoguanidine followed by 0 mM glyceraldehyde and in those pretreated with 8 mM aminoguanidine followed by 0, 1.5, and 2 mM glyceraldehyde were not significantly different. The aminoguanidine pretreatment completely inhibited the generation of intracellular TAGE in C2C12 cells treated with 1.5 and 2 mM glyceraldehyde (Fig. 1d and Additional file 1: Fig. S1b).

### Serum TAGE levels in STAM mice

**Fig. 2:**
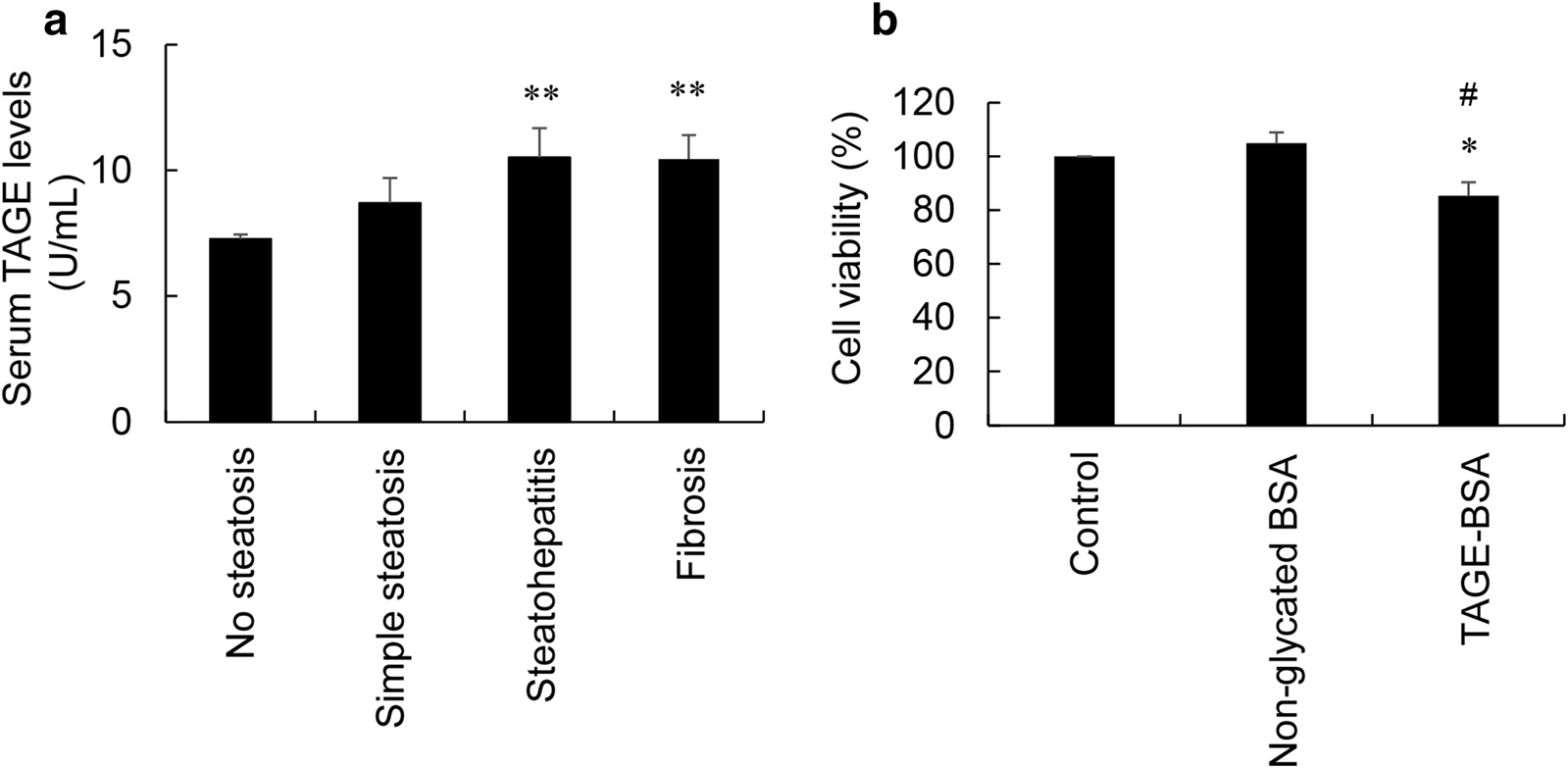
Serum TAGE levels in STAM mice and cytotoxicity of TAGE-BSA against C2C12 cells. **a** Serum TAGE levels in the four stage groups of STAM mice were measured using a competitive enzyme-linked immunosorbent assay. Results were expresses as TAGE units (U)/mL of serum, with 1 U corresponding to 1.0 μg of TAGE-BSA. There were 4 mice in each group. One experiment was performed using 4 wells against the serum of one mouse to calculate the average. Data are shown as mean ± S.D. (N = 4). P-values were based on the Bonferroni test. ^**^p < 0.01 vs. the no steatosis stage. **b** Cells were treated with 0 and 100 μg/mL non-glycated BSA and TAGE-BSA for 24 h. Cell viability was assessed by the WST-8 assay. This assay was performed in three independent experiments. One experiment was performed using 7 wells to calculate the average. Data are shown as mean ± S.D. (N = 3). P-values were based on the Bonferroni test. ^*^p < 0.05 vs. the control. ^#^*p *< 0.05 vs. the non-glycated BSA treatment

Serum TAGE levels in the no steatosis and simple steatosis stage groups were 7.27 ± 0.18 and 8.69 ± 1.01 U/mL, respectively (Fig. 2a). Serum TAGE levels in the steatohepatitis and fibrosis stage groups increased to 10.51 ± 1.16 and 10.44 ± 0.95 U/mL, which were higher than that in the no steatosis stage group.

### Viability of C2C12 cells treated with non-glycated BSA and TAGE-BSA

No significant differences were observed in the viability of C2C12 cells treated with 0, 20, and 50 μg/mL of non-glycated BSA and TAGE-BSA (Additional file 2: Fig. S2). The viabilities of C2C12 cells treated with 100 μg/mL non-glycated BSA and TAGE-BSA were 105.0 and 85.3%, respectively, and the ratio of cell viability was 81.2% (Fig. 2b).

## Discussion

Glyceraldehyde, which is a precursor of TAGE, is generated in the liver via three pathways [11, 15, 23]. (1) Glucose is metabolized to glyceraldehyde-3-phospate via glycolysis, and glyceraldehyde is produced by its dephosphorization through a non-enzymatic reaction. (2) Fructose is metabolized to glyceraldehyde via the pathway involving fructokinase and aldolase B (fructolysis). (3) Glucose is metabolized to fructose via the sorbitol pathway, which regulates aldose reductase and sorbitol dehydrogenase, and this fructose is metabolized to glyceraldehyde via fructolysis. Since skeletal muscle uses glycolysis and contains fructokinase, aldolase B, aldose reductase, and sorbitol dehydrogenase [26–28], we considered the three pathways of glyceraldehyde metabolism to occur in skeletal muscle, similar to the liver. CML and CEL are produced by some pathways from glucose [11, 15, 23], and CEL is produced by the degradation of the products of fructoselysine in glycated proteins [29].

CML- and CEL-modified proteins have been suggested to cause sarcopenia in C57Bl/6j mice fed a high-fat high-sugar diet and a high-fructose diet and also in ob/ob mice fed a standard diet based on the loss of muscle mass, myosteatosis, and oxidative stress in the gastrocnemius of these animals [9, 10]. However, the relationships between these AGEs and cell death in myoblasts remain unclear.

Therefore, we considered it important to investigate AGEs generated in myoblasts in order to clarify whether the mechanisms contributing to the loss of skeletal muscle involve the death of or dysfunctions in myoblasts. Although CML- and CEL-modified proteins may be generated in myoblasts, we hypothesized that TAGE are produced by these cells and induce cytotoxicity based on previous findings showing their production by neuroblastoma cells, hepatic cells, pancreatic cells, and cardiac cells and induction of cell death [16–22].

We treated C2C12 cells with glyceraldehyde to rapidly generate intracellular TAGE. In the present study, C2C12 cells were treated with glyceraldehyde at a physiological concentration to generate TAGE within 24 h. Taniguchi et al. [30] previously demonstrated that islets of the pancreas exposed to 20 mM glucose accumulated 0.025 pmol/islet glyceraldehyde, whereas exposure to 10 mM glyceraldehyde caused the accumulation of 0.12 pmol/islet glyceraldehyde. Based on these findings, Takahashi et al. [31] used 2 mM glyceraldehyde in their experiments, which is a similar concentration to 20 mM glucose. On the other hand, plasma levels of glucose in NASH and T2DM model mice increased by more than 25 mM [32–34]. The viability of C2C12 cells treated with 1.5 and 2 mM glyceraldehyde for 24 h dose-dependently decreased (Fig. 1a). In contrast, intracellular TAGE were generated in a dose-dependent manner (Fig. 1b and Additional file 1: Fig. S1a). To demonstrate that the generation of TAGE decreased cell viability, C2C12 cells were pretreated with 8 mM aminoguanidine, an inhibitor of the generation of AGEs, for 2 h followed by 1.5 and 2 mM glyceraldehyde for 24 h. Aminoguanidine inhibited decreases in cell viability as well as the generation of TAGE (Fig. 1c, d and Additional file 1: Fig. S1b). To the best of our knowledge, this is the first study to show that intracellular TAGE were generated from glyceraldehyde at a physiological concentration in myoblasts and strongly induced cell death. The death of myoblasts will lead to the loss of skeletal muscle. Living myoblasts that generate intracellular TAGE may also ultimately lead to the loss of skeletal muscle. In our previous study, when rat primary cardiomyocytes were treated with 4 mM glyceraldehyde for 6 h, cell viability decreased to 39.2% and intracellular TAGE were generated at 12.0 μg/mg protein [22]. Furthermore, living cardiomyocytes completely stopped beating. The viability of C2C12 cells treated with 1.5 mM glyceraldehyde was 47.7% and living cells generated intracellular TAGE levels of 6.0 μg/mg protein (Fig. 1a, b and Additional file 1: Fig. S1a). The generation of skeletal muscle may be inhibited in myotubes with dysfunctional differentiation [2–6]. Collectively, these findings and the present results suggest that the cell death or dysfunction of myoblasts that gain excess glucose or fructose and generate high levels of intracellular TAGE may inhibit the differentiation of myoblasts.

TAGE in the blood which are one of extracellular TAGE increase in patients with LSRD and induce responses such as inflammation and oxidative stress in cells via RAGE [11–13, 15, 23]. Extracellular TAGE which are generated in other cells and secreted or released into the blood may induce cytotoxicity in myoblasts because they express RAGE [2]. Therefore, we investigated the cytotoxicity of TAGE-BSA, a model of extracellular TAGE, in C2C12 cells. We considered that the concentration of TAGE-BSA should be decided based on the physiological concentration of TAGE in the blood of LSRD model mice. Since we previously reported that serum TAGE levels were higher in NASH patients than in healthy controls and patients with simple steatosis [14], we measured serum TAGE levels in STAM mice, a NASH model. Serum TAGE levels were approximately 1.4-fold higher in the steatohepatitis and fibrosis stages than in the no steatosis stage, and ranged between approximately 7 and 11 U/mL (Fig. 2a). Although we did not measure serum TAGE levels in other LSRD model mice, we speculate that they may not differ markedly range between 7 and 12 U/mL based on the data of serum TAGE levels of many patients with LSRD such as NASH, T2DM, and CVD [14, 25, 35, 36]. We applied 20, 50, and 100 μg/mL TAGE-BSA, which are approximately 2, 5, and tenfold, respectively, that of serum TAGE levels in STAM mice that develop steatohepatitis and fibrosis (Fig. 2 and Additional file 2: Fig. S2). To examine the effects of TAGE in C2C12 cells, we assessed the viability of C2C12 cells treated with non-glycated BSA and TAGE-BSA. Only 100 μg/mL TAGE-BSA slightly decreased cell viability (Fig. 2b). Therefore, extracellular TAGE do not appear to induce cell death under physiological conditions.

## Conclusion

The present study demonstrated that intracellular TAGE were generated in C2C12 cells and more strongly induced cell death than extracellular TAGE. Therefore, intracellular TAGE may induce cell death in the myoblasts of LSRD model mice.

## Supplementary information

Supplementary material 1 (DOC 369 kb)

Supplementary material 1 (DOC 232 kb)

## Data Availability

The datasets used and/or analyzed during the present study are available from the corresponding author upon reasonable request.

## Abbreviations

LSRD: Lifestyle-related diseases
AGEs: Advanced glycation end-products
TAGE: Toxic advanced glycation end-products
T2DM: Type 2 diabetes mellitus
NASH: Non-alcoholic steatohepatitis
CML: *N*^ε^-carboxymethyllysine
CEL: *N*^ε^-carboxyethyllysine
CVD: Cardiovascular disease
