# Supplementary figures and images for: Impact of intracellular toxic advanced glycation end-products (TAGE) on murine myoblast cell death
Source: Diabetol Metab Syndr. 2020 Jun 29;12:54. doi: 10.1186/s13098-020-00561-z (PMC7362572; doi:10.1186/s13098-020-00561-z)

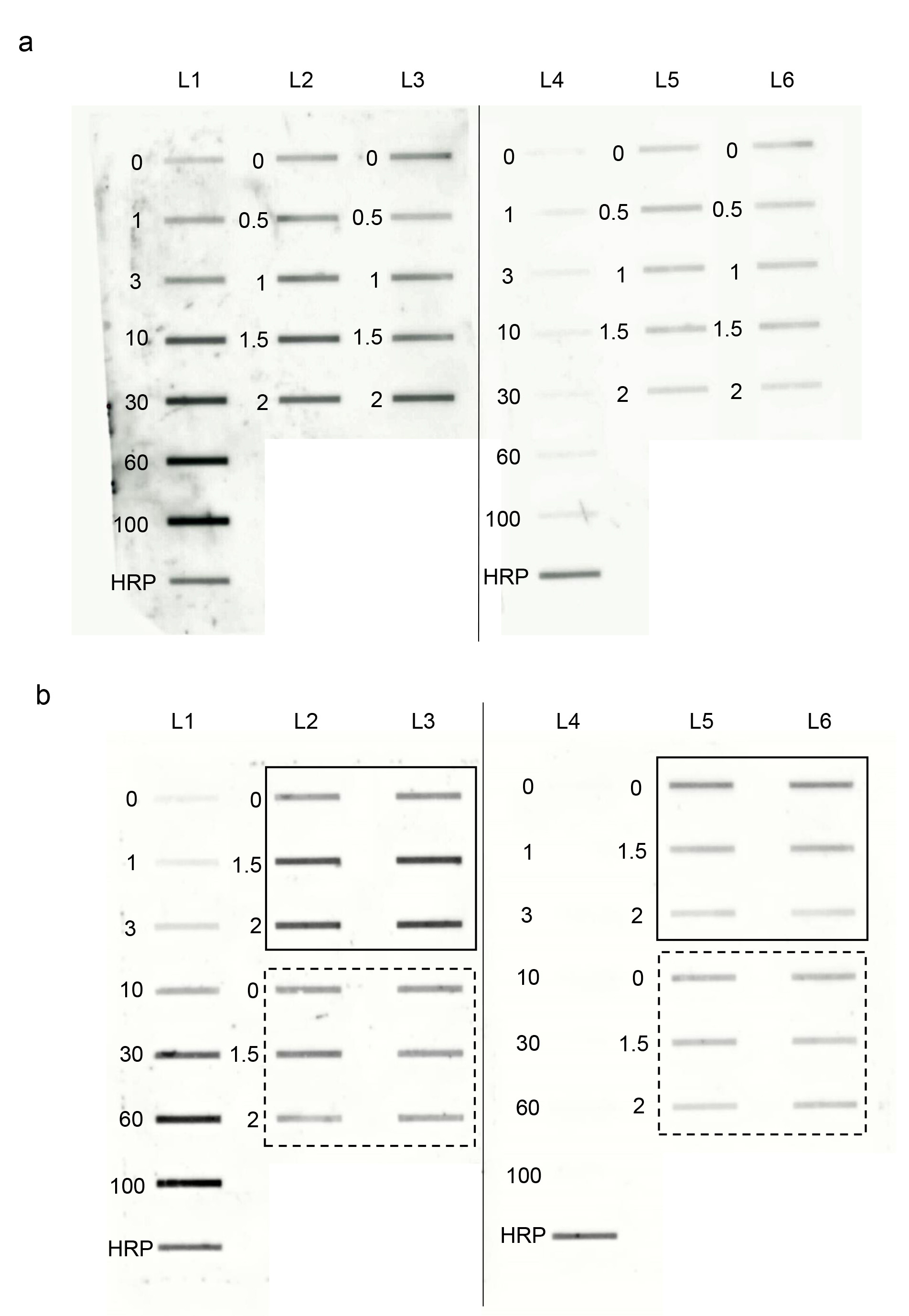

Supplement: Supplementary file 1 — Supplementary material 1 (DOC 369 kb) [file 13098_2020_561_MOESM1_ESM.jpg]

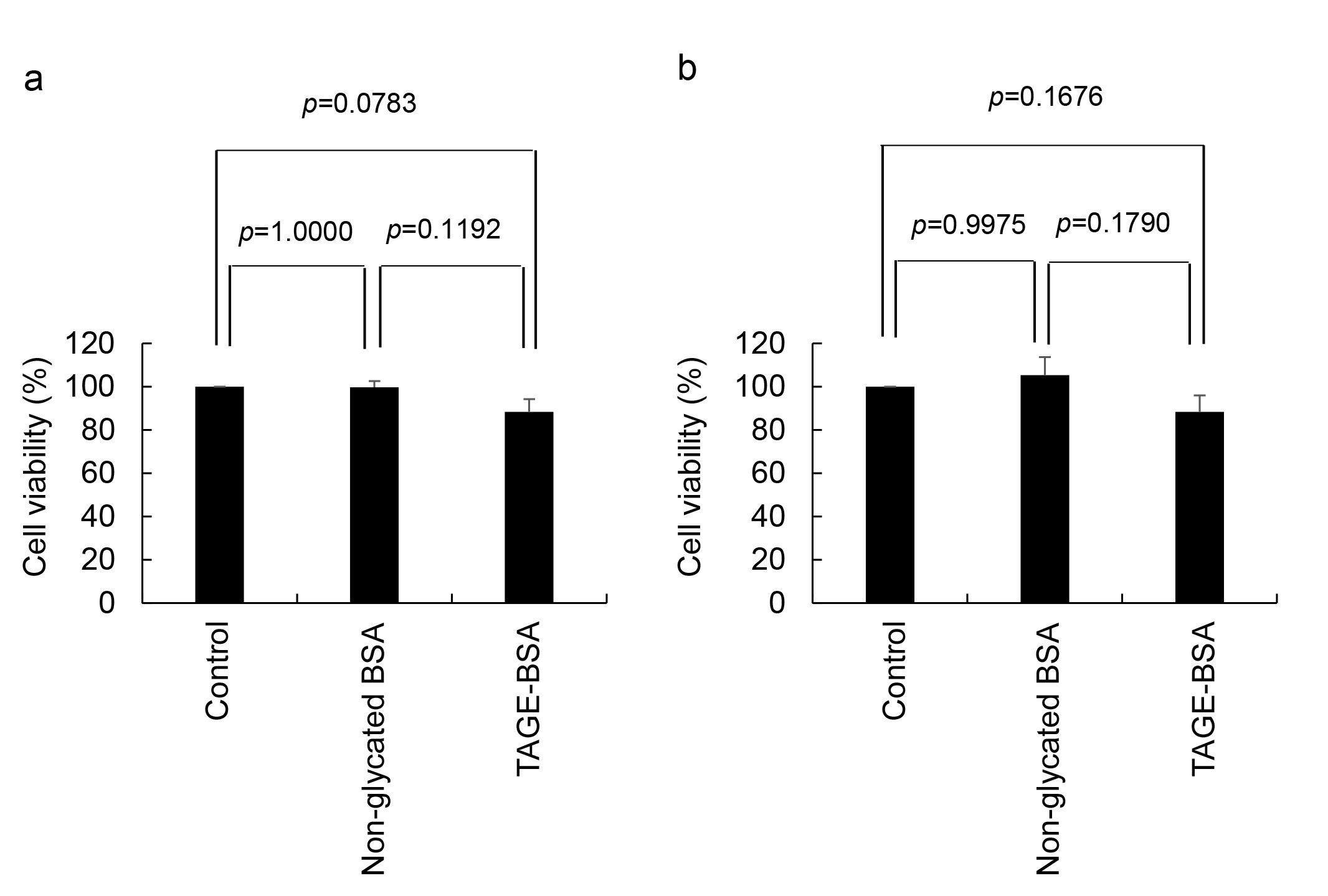

Supplement: Supplementary file 2 — Supplementary material 1 (DOC 232 kb) [file 13098_2020_561_MOESM2_ESM.jpg]
